# Optimizing contrast timing in photon-counting detector CT for the assessment of peripheral arthritis

**DOI:** 10.1007/s00256-025-04993-4

**Published:** 2025-08-08

**Authors:** Akos Horvath, Rita Csercsik, Janos Norbert Gyebnar, Daniel Sandor Veres, Pal Maurovich-Horvat, Akos Menyhart, Viktoria Gyergyoi, Peter Vince Balint, Nikolett Marton

**Affiliations:** 1 Department of Radiology, Clinic for Medical Imaging, Semmelweis University, 2. Koranyi Sandor Utca, Budapest, 1083 Hungary; 2 Department of Biophysics and Radiation Biology, Semmelweis University, Budapest, Hungary; 3 Department of Rheumatology, Clinic for Rheumatology and Immunology, Semmelweis University, Budapest, Hungary

**Keywords:** Photon-counting detector CT, Spectral CT, Iodine mapping, Peripheral arthritis, Rheumatoid arthritis, Psoriatic arthritis, Synovitis, Tenosynovitis, Hand, Wrist

## Abstract

**Objective:**

Photon-counting detector CT (PCD-CT) allows for iodine mapping of inflamed tissues in peripheral immune-mediated arthritis, supporting diagnosis and disease activity assessment. This study aims to identify the optimal timing for image acquisition after intravenous iodinated contrast administration to maximize enhancement and contrast with surrounding tissues.

**Methods:**

High-resolution PCD-CT scans of bilateral wrist–hand regions were obtained from 26 patients with peripheral arthritis, both native and post-contrast (1 ml/kg intravenous iodinated contrast at 2.5 ml/sec flow) at 120-, 180-, and 240-s delay phases. Iodine maps were constructed from spectral data. Phases were compared based on densities and iodine concentrations measured in synovial, tenosynovial, and periungual tissues, with muscle, fat, and vessels as controls. We used descriptive statistics and mixed-effects linear regression inferential models for the comparisons. Synovitis and tenosynovitis were verified by ultrasound measurements.

**Results:**

No significant differences (*p* > 0.05) were found in iodine concentration or density across the 120-, 180-, and 240-s post-contrast phases in inflamed synovial, tenosynovial, and periungual soft tissues. Inflamed tissues showed significant and consistent differences in iodine concentration from muscle and fat (*p* < 0.0001) across all phases, while the greatest differentiation from vessels was in the 120-s phase. The effective dose was identical across all post-contrast phases (0.028 ± 0.0035 mSv).

**Conclusion:**

Iodine uptake in inflamed tissues was identical across all three post-contrast phases. However, the 120-s phase offered the highest contrast between inflammation and surrounding vascular structures while minimizing scan time, supporting its use for standardized follow-up imaging.

**Supplementary Information:**

The online version contains supplementary material available at 10.1007/s00256-025-04993-4.

## Introduction

Immune-mediated peripheral arthritis commonly affects the small joints of the hands and feet. Early inflammatory activity—such as synovitis, tenosynovitis, tendinitis, or enthesitis—can lead to irreversible structural damage if left untreated [1, 2]. Since therapeutic efficacy is highest when disease-modifying antirheumatic drugs (DMARDs) are initiated early, sensitive imaging modalities are essential for timely diagnosis and monitoring of treatment response [3, 4].

MRI and ultrasound are most commonly used for the assessment of inflammatory activity in peripheral arthritis. However, due to its relatively low specificity, MRI is now primarily used in clinical trials and is no longer the modality of first choice [5]. In addition to its limited spatial resolution, low availability, and high cost, MRI also tends to produce motion artifacts in the distal phalanges. Ultrasound offers better spatial resolution and greater accessibility, but it is depth limited and highly operator dependent.

Computed tomography (CT) has historically played a limited role in arthritis imaging due to concerns about radiation exposure and poor iodine contrast resolution. However, the advent of spectral imaging—particularly with dual-energy CT and photon-counting detector CT (PCD-CT)—has markedly improved its diagnostic utility.

PCD-CT enables high-resolution imaging and iodine mapping at reduced radiation doses. Iodine maps can quantify contrast uptake and visualize hypervascularity in inflamed tissues, thereby objectively monitoring disease activity. They offer high spatial resolution with fewer motion artifacts, allowing reliable detection of soft tissue inflammation even in anatomically complex regions such as the distal interphalangeal joints [6]. Compared to contrast-enhanced MRI, iodine maps demonstrate superior sensitivity for detecting inflammatory lesions and may support early differentiation of inflammatory arthritides by depicting the ultrastructural distribution of inflammation [6, 7].

In addition to soft tissue assessment, CT has a very high sensitivity for detecting osseous changes, particularly early erosions that signal disease progression. Its shorter scan times compared to MRI and US also improve patient comfort. By enabling evaluation of both inflammatory activity and structural damage, PCD-CT provides a comprehensive and patient-friendly approach to imaging peripheral arthritis.

Despite its promise, limited data are available regarding optimal imaging protocols for iodine mapping in peripheral arthritis. In particular, the ideal post-contrast acquisition timing for balancing tissue enhancement and soft tissue contrast remains undefined.

Our aim was to determine the optimal timing for iodine-enhanced PCD-CT in the evaluation of peripheral arthritis. We sought to identify the delay phase that provides the highest enhancement of inflammation and best distinguishes inflammatory lesions from adjacent structures.

## Methods

### Study population

**Fig. 1:**
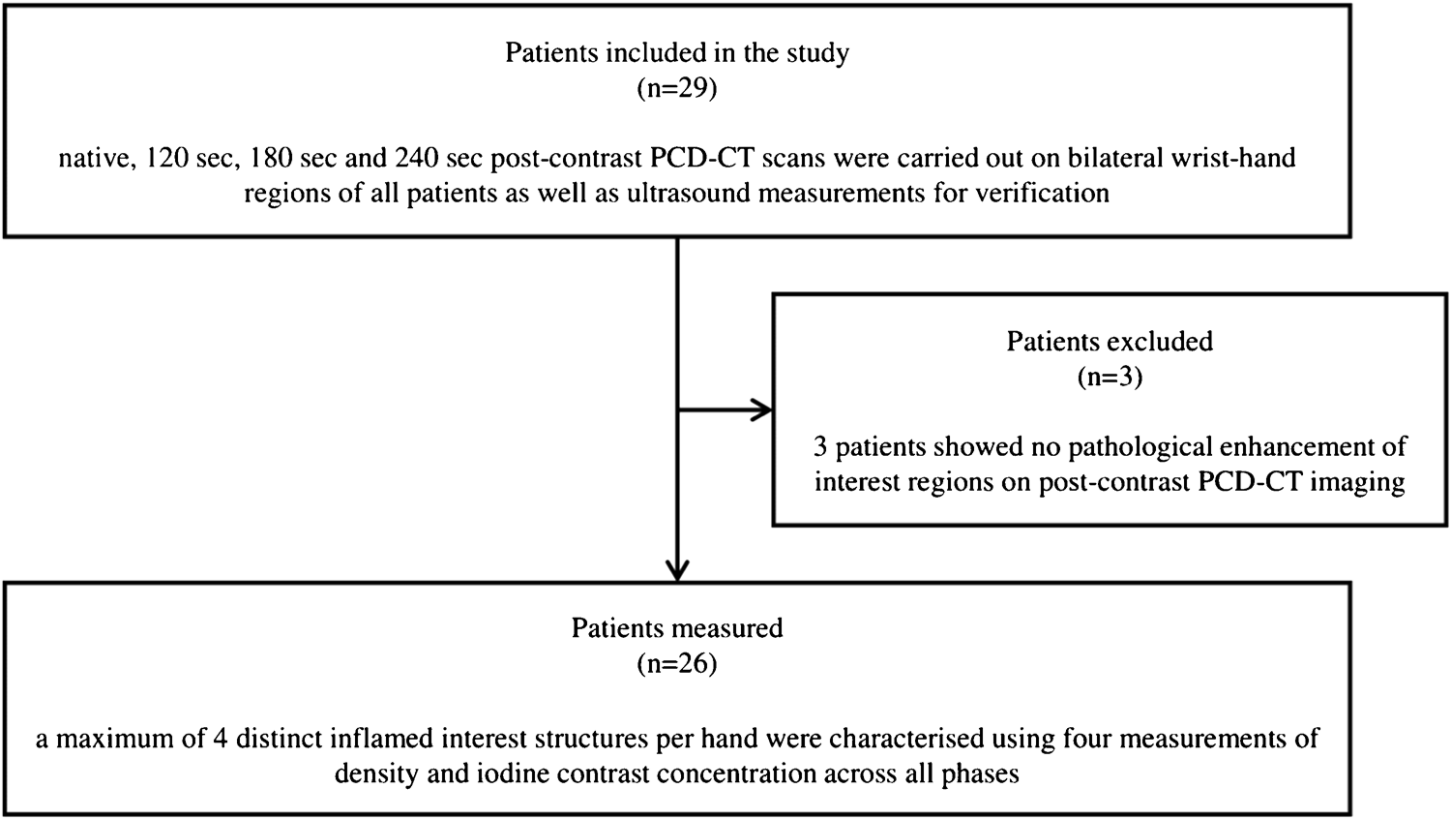
The flowchart outlines the patient selection process. A total of 29 patients with peripheral arthritis were enrolled and underwent PCD-CT examinations. Three patients were excluded due to the absence of pathological enhancement (no definite, unambiguous synovitis, tenosynovitis, or periungual soft tissue inflammation) on the post-contrast images. PCD-CT, photon counting detector computed tomography

Twenty-nine patients with peripheral arthritis were prospectively recruited for the study. Diagnoses were made by board-certified rheumatologists based on clinical, laboratory, and imaging findings. Rheumatoid arthritis was diagnosed using the 2010 ACR/EULAR classification criteria and psoriatic arthritis using the CASPAR criteria [8, 9]. Seronegative spondyloarthritis was defined by the Assessment of SpondyloArthritis international Society classification criteria [10]. Calcium pyrophosphate deposition disease was diagnosed based on the 2023 ACR/EULAR classification criteria [11]. Exclusion criteria were age younger than 18 years, pregnancy, previous hypersensitivity reaction to iodine-based intravenous CT contrast media, glomerular filtration rate < 40 ml/min, untreated hyperthyroidism, and inability to give informed consent. Additionally, three patients were excluded due to the absence of pathological enhancement on contrast-enhanced PCD-CT scans in any synovial, tenosynovial, or periungual soft tissue structures in the investigated areas (Fig. 1). Imaging measurements were conducted between February 21, 2023, and April 30, 2024. The demographic and clinical data of the study participants were recorded (Table 1). All patients were monitored regularly and received medical treatment as recommended by their attending rheumatologist, following standards of care. Written informed consent was obtained from all study participants.

**Table 1:** Patient characteristics

Demographics	
Number of patients	26
Age (years) (mean ± standard deviation)	52.58 ± 18.14
Gender (*n*, %)	
Female	20 (77%)
Male	6 (23%)
Ever smoker (*n*, %)	2 (7.69%)
Disease onset (years) (mean ± standard deviation)	5.38 ± 6.27
Diagnostics (ACR/EULAR, CASPAR) (*n*, %)	
Seropositive rheumatoid arthritis	9 (34.62%)
Seronegative rheumatoid arthritis	6 (23.08%)
Psoriatic arthritis	6 (23.08%)
Seronegative spondyloarthritis	2 (7.69%)
Calcium pyrophosphate deposition disease	1 (3.85%)
Polyarthralgia	1 (3.85%)
Suspected polymyalgia rheumatica	1 (3.85%)
Laboratory findings	
CRP (mg/l) (mean ± standard deviation)	10.85 ± 13.1
ESR (mm/h) (mean ± standard deviation)	23.09 ± 16.9
Severity index (DAS28) (mean ± standard deviation)	3.86 ± 1.06
Therapy (*n*, %)	
None	11 (42.31%)
Methotrexate	9 (34.62%)
Steroid	2 (7.69%)
Sulfasalazine	1 (3.85%)
Celecoxib	1 (3.85%)
Leflunomide	1 (3.85%)
Hydroxychloroquine	1 (3.85%)

*ACR *American College of Rheumatology, *CRP *Creactive protein, *DAS28 *disease activity score, *ESR *erythrocyte sedimentation rate, *EULAR *European Alliance of Associations for Rheumatology

### Radiological examinations and post-processing

High-resolution PCD-CT scans (NAEOTOM Alpha Peak, Siemens Healthineers) with a 0.4-mm slice thickness and 0.85-mm pitch were obtained for bilateral wrist–hand regions of all patients in four phases: without contrast and with intravenous iodinated contrast at delays of 120, 180, and 240 s (sec). During the scans, patients lay prone with both their arms outstretched and pronated, with only their hands positioned inside the gantry. Then, 1 ml/kg of body weight of iodinated contrast was administered through the cubital vein at a flow rate of 2.5 ml/sec. We used Iomeron 350 and Ultravist 370, both non-ionic, low-osmolar iodinated contrast agents, with iodine concentrations of 350 and 370 mg/ml, respectively. The scans were performed with an X-ray tube voltage of 140 kV, producing an average X-ray tube current of 39.54 ± 3.06 mA (mean ± standard deviation) for the native series and 49.04 ± 3.33 mA (mean ± standard deviation) for the post-contrast series. Spectral images were post-processed from raw data to create iodine maps. First, virtual non-enhanced images were generated from the post-contrast images. A color map, created from the iodine concentration values calculated from the spectral data, was then overlaid onto the non-enhanced images. The color maps were normalized to radial arteries.

On each hand of every patient, up to four of the most intensely enhancing inflamed synovial, tenosynovial, or periungual soft tissue structures were selected as target ("interest") structures. Periungual soft tissues were included based on prior studies suggesting that inflammation at the nail bed enthesis—particularly in psoriatic arthritis—may represent an early and disease-specific manifestation [6]. Within each selected structure, density and iodine concentration values were measured manually at four distinct measurement points using circular regions of interest (ROIs). Density measurements were taken from virtual non-enhanced images, while iodine concentration values were extracted from the iodine maps. For joints and tendons, ROIs were placed in areas of thickened synovium or tenosynovium, while for periungual tissues, ROIs were placed in the most homogeneously enhancing soft tissue regions.

**Fig. 2:**
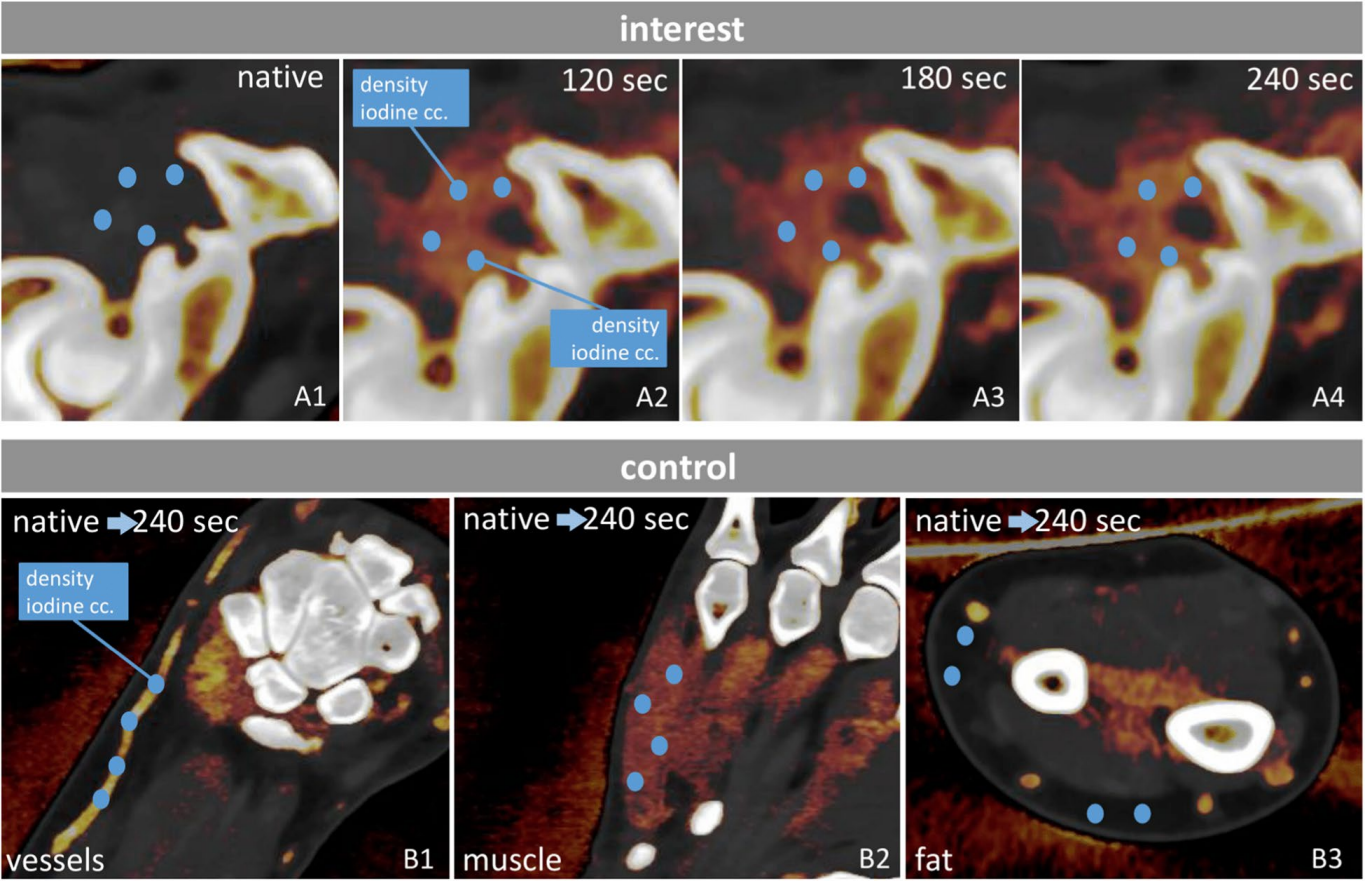
Chart of our measurement process. Four measurement points (dark blue circles) were selected for each of the maximum four most enhancing synovial and soft tissue structures. Density and iodine concentration values were registered at each measurement point. The same measurement points were then assessed on every phase (native; 120 s, 180 s, 240 s post-contrast; **A1–A4**). For control, vessels, muscle tissue, and fat tissue samples were measured in the same way for each side (**B1–B3**, different color map windowing). cc., concentration; sec, second

For each scan phase (native, 120 s, 180 s, and 240 s), the same four interest structures were evaluated, with ROI locations kept consistent across phases (Fig. 2, A1–A4). In total, 492 interest measurement points were assessed per phase.

For control, enhancing vessels, muscle tissue, and fat tissue samples were chosen, as they are the most frequent types of tissues surrounding inflamed structures. Muscles and vessels often show comparable iodine concentrations to inflamed structures; thus, examining contrast differentiation is relevant. Control structures were measured in the same way as interest structures. ROIs for muscle were placed in homogeneously enhancing thenar and hypothenar regions, fat ROIs were positioned in subcutaneous fat around the wrist, and vessel ROIs were placed in proximal enhancing arteries near the wrist and metacarpals (Fig. 2, B1–B3). This yielded 564 control measurement points per phase.

Measurements were performed in anatomical planes that offered the clearest visualization of the target structures, taking advantage of CT’s multiplanar reconstruction capabilities. These planes were kept consistent across all phases. ROI placement consistency was ensured by visual alignment of anatomical landmarks, which was necessary due to minor patient hand movements between phases. Consistency was further validated by comparing density values across corresponding ROIs in different phases.

ROI placement and data recording were carried out by the lead author, an undergraduate research student with 3 years of experience in wrist–hand CT imaging, and a board-certified radiologist with a musculoskeletal imaging focus and 9 years of experience. Inter-reader agreement was calculated to assess reproducibility.

Image post-processing and measurements were performed using Siemens Healthineers’syngo.via software, and the data were recorded in spreadsheets. The density and iodine concentration values were compared across the native and three post-contrast phases and were controlled using our control sample measurements. The relation between interest and control variables was also analyzed.

All cases of synovitis and tenosynovitis detected on CT were verified by ultrasound using a 10–15-MHz high-frequency linear transducer (RS85A; Samsung). Ultrasound examinations were performed by a board-certified radiologist with 7 years of experience. Synovial and tenosynovial thickening, joint effusion, and increased vascularity were considered indicative of inflammation, based on OMERACT definitions [12].

### Ethical considerations

This trial was approved by the ethics review board. Informed consent was obtained from all individual participants included in the study. This work was carried out in accordance with the Helsinki Declaration (JAMA 2000; 284:3043–3049).

### Statistics

For the evaluation of patient characteristics, imaging protocol data, and dose values, descriptive statistics were used. For statistical inference, mixed-effects linear regression models were employed, with density as the target variable in one model and iodine concentration in the other. Localization (categorized as interest, vessel, muscle, and fat) and measurement time (as a categorical variable for the four phases) were included as explanatory variables, along with their interactions. Random-effects intercepts were included in the hierarchical model for patients and the examined body side. Model diagnostics, based on normalized residuals (linearity, heteroscedasticity, and normality), were acceptable for both models. Confidence intervals for comparing the levels of the explanatory variables (using interest measurements as the reference) were calculated with Sidak’s correction method. All statistical analyses were made with *R* statistical software [13] (v4.4.1) using the Table 1 package for descriptive tables [14] (v1.4.3), the *ggplot2* package for descriptive plots [15] (v3.5.1), and the *nlme* [16] (v3.1.165) and *emmeans* [17] (v1.10.2) packages for regression model calculations and interpretations. Intraclass correlation coefficients (ICCs) were calculated to analyze the possible correlation between the two raters. For decisions, we used 5% as significance level.

## Results

### Comparison of iodine enhancement patterns across post-contrast phases

**Fig. 3:**
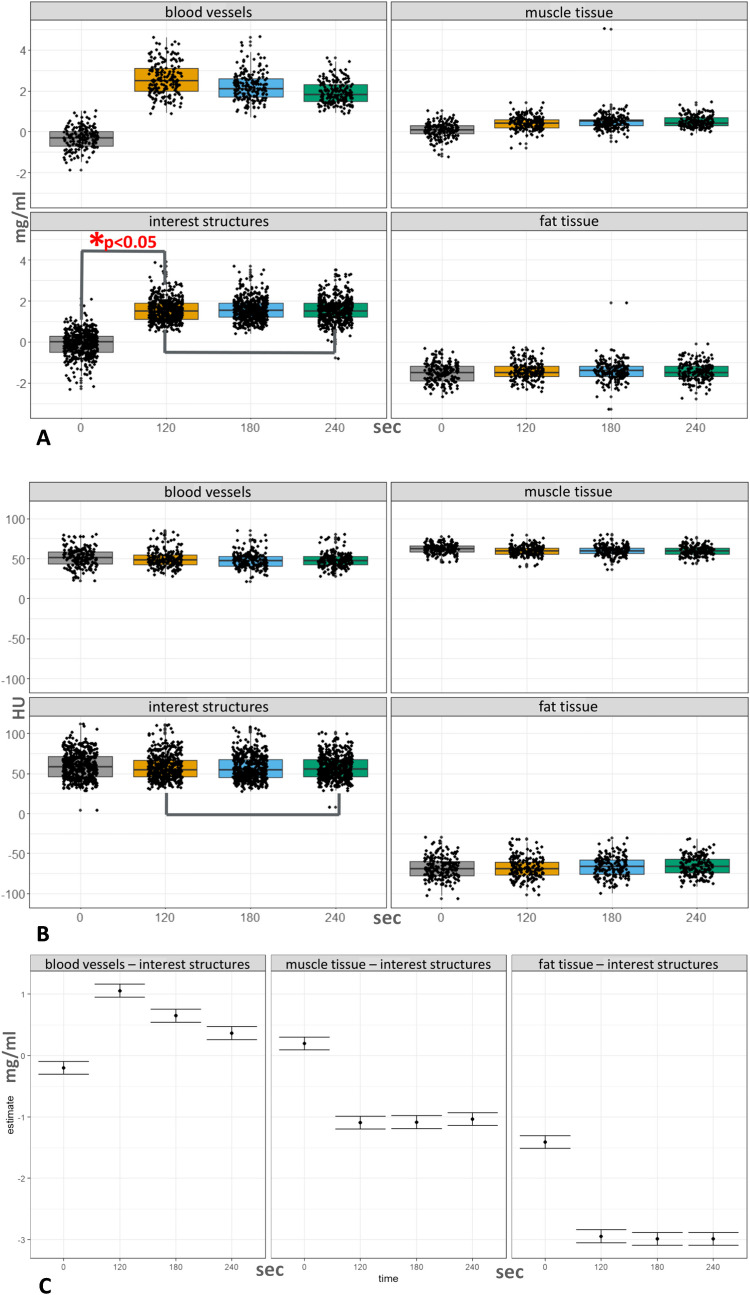
Results.** A**–**B** compares the density and iodine concentration values of interest structures as well as control structures between the investigated time points (individual values and box plots showing the quartiles). **C** demonstrates the differential enhancement of interest structures compared to control tissue samples (estimated difference with 95% confidence interval). sec, second

No statistically significant (*p* > 0.05) difference was observed in the mean iodine concentration values of the synovial, tenosynovial, and periungual soft tissue structures of interest between the 120, 180, and 240 s post-contrast phases (Supplementary Table 1). The mean iodine concentration values (mg/ml ± standard deviation) of interest structures in the native, 120 s, 180 s, and 240 s phases were 1.54 (± 0.552), 1.58 (± 0.534), and 1.54 (± 0.574), respectively. There was a statistically significant difference (*p* < 0.0001) in the mean iodine concentrations of interest structures between the native and post-contrast phases, with the mean concentration value (mg/ml) for interest structures in the native phase being − 0.098 (Fig. 3A).

Control muscle tissue samples showed a slight increase in enhancement with each successive phase from native to 240 s post-contrast, with mean concentration values (mg/ml ± standard deviation) in the native, 120 s, 180 s, and 240 s phases being 0.084 (± 0.354), 0.430 (± 0.331), 0.483 (± 0.446), and 0.493 (± 0.280), respectively. In contrast, fat tissue samples exhibited nearly constant mean concentration values, concentrations (mg/ml) in the native, 120 s, 180 s, and 240 s phases being − 1.52 (± 0.479), − 1.42 (± 0.439), − 1.42 (± 0.499), and − 1.46 (± 0.430), respectively. Control blood vessels demonstrated early enhancement, then gradual washout, with mean concentration values (mg/ml ± standard deviation) in the native, 120 s, 180 s, and 240 s phases being − 0.315, 2.58, 2.22, and 1.89, respectively (Fig. 3A).

All interest and control structures maintained the same mean density across the native and the three post-contrast phases (Fig. 3B).

To analyze inter-reader agreement between the two readers, intraclass correlation was calculated. For the density values, the agreement was excellent, ICC: 0.983 (confidence interval: 0.983–0.988). For the concentration data, the agreement was good, ICC: 0.855 (confidence interval: 00.736–0.9735).

Regarding the relation between enhancing interest structures and control tissues, our mixed-effects linear regression models demonstrated significantly different iodine concentrations in enhancing interest structures compared to all three types of control tissue samples across all phases (*p* < 0.0001). The greatest difference in concentration between interest structures and blood vessels was observed at the 120-s phase, with each subsequent phase showing a gradual decrease in this difference [estimated mean concentration difference (mg/ml) in the 120-, 180-, and 240-s phases, respectively: 1.055, 0.649, 0.364]. This highlights the greatest contrast between vessels and interest structures at 120 s. The concentration difference between interest structures and muscle [estimated mean concentration difference (mg/ml) in the 120-, 180-, and 240-s phases, respectively: 1.094, 1.086, 1.035] and interest structures and fat [estimated mean concentration difference (mg/ml) in the 120-, 180-, and 240-s phases, respectively: 2.945, 2.988, 2.987] remained nearly constant across all post-contrast phases (Fig. 3C, Supplementary Tables 2 and 3). Fat tissue was used as a negative control for contrast enhancement. Due to technicalities in the way the syngo.via software calculates iodine concentrations, fat tissue was interpreted as having negative iodine concentration values, a phenomenon previously reported by Pelgrim et al. in dual-energy CT systems [18].

A comparison between diagnostic groups was performed, but no significant differences were found in the density or concentration values among the various arthritis types (Supplementary Table 4).

No correlation with clinical data (age, disease onset, CRP levels, erythrocyte sedimentation rates, severity index scores) was found (Supplementary Table 5).

### Distribution of contrast-enhancing areas in the investigated hand–wrist regions

Among our patients, the most commonly affected and measured inflamed structures were the radio- and ulnocarpal joints, the proximal interphalangeal (PIP) joints and metacarpophalangeal (MCP) joints of the first three digits, as well as digital flexor tendons, the extensor carpi ulnaris (ECU) tendon, the distal radioulnar (DRU) joint, and periungual soft tissues. Table 2 lists all of the assessed structures and the associated number of measurement points within those structures per phase across all patients.

**Table 2:** Measurement locations and the associated number of measurement points (*n*) per phase across all patients

Joints	*n*
Radiocarpal joint	52
III PIP joint	44
Ulnocarpal joint	40
I MCP joint	24
II MCP joint	24
II PIP joint	20
DRU joint	20
I CMC joint	16
III MCP joint	12
Intercarpal joint	12
II DIP joint	8
III DIP joint	8
Pisotriquetral joint	8
STT joint	8
V MCP joint	4
I IP joint	4
IV DIP joint	4
Triquetrohamate joint	4
Tendons	*n*
II digit flexor tendon	40
ECU tendon	20
III digit flexor tendon	16
II digit extensor tendon	8
ECRB tendon	8
ECRL tendon	4
III digit extensor tendon	4
IV digit flexor tendon	4
FCU tendon	4
Periungual soft tissues	*n*
II digit PST	20
III digit PST	20
IV digit PST	12

*CMC* carpometacarpal, *DIP* distal interphalangeal, *DRU* distal radioulnar, *ECRL* extensor carpi radialis longus, *ECRB* extensor carpi radialis brevis, *ECU* extensor carpi ulnaris, *FCU* flexor carpi ulnaris, *IP* interphalangeal, *MCP* metacarpophalangeal, *PIP* proximal interphalangeal, *PST* periungual soft tissue, *STT* scaphotrapeziotrapezoidal

### Dose considerations

PCD-CT total dose length product (tDLP) values were extracted from patient protocol data using Siemens Healthineers’ syngo.via software. The average tDLP value was 105.06 ± 15.38 mGy*cm (mean ± standard deviation) for the native series and 140.81 ± 17.3 mGy*cm (mean ± standard deviation) for each of the post-contrast series. The average tDLP per patient was 549.88 ± 89.4 mGy*cm (mean ± standard deviation). Effective dose values were calculated using the formula: effective dose = tDLP * conversion factor. Using a body region–, age-, and X-ray tube voltage–specific conversion factor of 0.0002 mSv*mGy^−1^*cm^−1^, specific to extremities, adults, and an X-ray tube voltage of 140 kV, the average total effective dose value per patient was estimated to be 0.11 ± 0.02 mSv (mean ± standard deviation) [19]. The effective dose for each post-contrast phase was 0.028 ± 0.0035 mSv (mean ± standard deviation), demonstrating no relevant difference in radiation burden between the three investigated post-contrast phases.

## Discussion

With the advent of biologics, early detection of synovitis and tenosynovitis in peripheral arthritis, such as rheumatoid arthritis and psoriatic arthritis, has become increasingly important [20]. By the detection of subclinical inflammation and early treatment initiation, the development of irreversible structural changes can be prevented [21]. However, imaging standardization and reliable exclusion of differential diagnoses are required.

CT provides high spatial resolution and thin-slice multiplanar reconstructions, making it well suited for evaluating small, distal structures such as the DIP joints, nailbeds, and their ligamentous and enthesial complexes—common sites of involvement in psoriatic arthritis [6]. This rationale underpins our inclusion of periungual soft tissues in the analysis. Inflammation in PsA primarily targets the synovio-entheseal complex, including the extensor tendon insertion at the nailbed, where it is linked to psoriatic nail changes—often the first or only sign of the disease [22–24]. Because this inflammation frequently extends into adjacent soft tissues, periungual regions are important to assess [25]. CT also provides more detailed information about bones and calcified tissues. CT is considerably more sensitive than MRI and ultrasound when it comes to the detection of early erosions, a hallmark of disease progression in erosive arthritis [20].

**Fig. 4:**
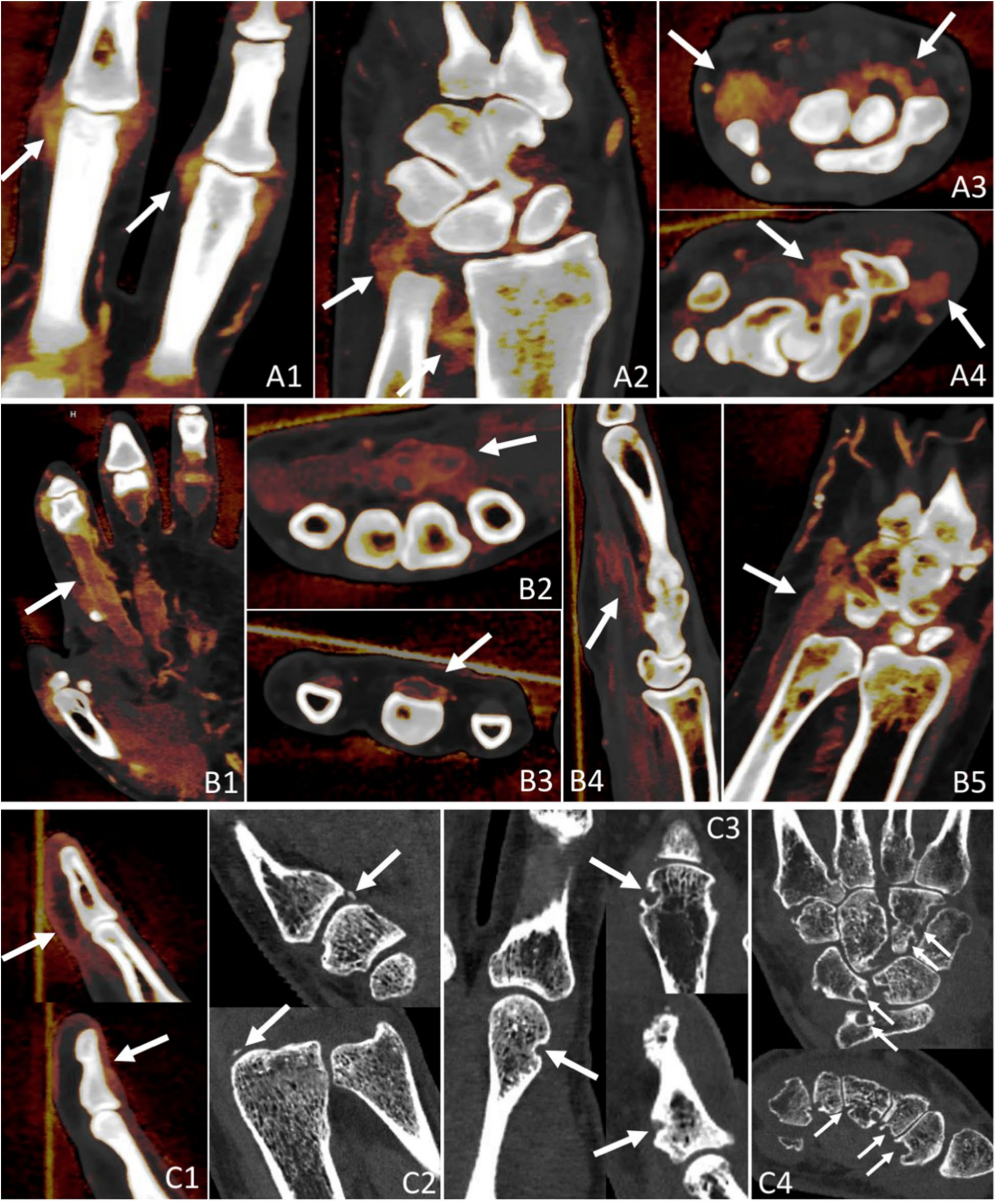
Typical findings in inflammatory peripheral arthritis seen on our PCD-CT images. PCD-CT iodine maps provide high iodine contrast, accurately delineating inflammation, while also displaying bones with high resolution. Synovitis appears as areas of high iodine concentration, represented in red, reflecting thickened and hypervascular synovium, as demonstrated in images **A1**–**A4**. This thickening is often nodular due to pannus formation, as seen in **A3** (right arrow). Tenosynovitis exhibits similar imaging characteristics to synovitis, with high iodine concentration indicating inflammation, as shown in **B1**–**B5**. Periungual soft tissue inflammation, characteristic of psoriatic arthritis, is evident on PCD-CT iodine maps, as demonstrated in **C1**. In this particular case, the inflammation was detected exclusively on PCD-CT, as neither ultrasound nor MRI identified it. Periostitis, appearing as juxtaarticular new bone formation, also characteristic of psoriatic arthritis, is visible on PCD-CT images (**C2**), and this finding was also uniquely detected with PCD-CT. Cortical bony erosions of varying shapes and sizes, indicative of longstanding arthritis, are well demonstrated in **C3** and **C4**. CT remains the most sensitive imaging modality for detecting erosions, which serve as a critical marker of disease progression. MRI, magnetic resonance imaging; PCD-CT, photon-counting detector CT

Spectral CT is an advanced imaging technique that is gaining increasing prominence in musculoskeletal imaging. Spectral data can be acquired using dual-energy CT (DE-CT) or multienergy photon-counting detector CT systems. Using this spectral data, iodine maps can be constructed, which provide much better iodine contrast resolution compared to conventional contrast-enhanced CT images at higher spatial resolutions. They quantitatively reveal the presence of iodine in synovial and tenosynovial tissues, serving as a marker of hypervascularization often associated with inflammatory activity (Fig. 4).

Ulas et al. demonstrated that iodine mapping has similar sensitivity and specificity to contrast-enhanced MRI in the detection of synovitis and tenosynovitis in arthritis of the hand [26]. Paired with high spatial resolution, DE-CT can differentiate between various inflammatory patterns in different arthritis, aiding in the early selection of the appropriate treatment [6]. Additionally, through material decomposition techniques, DE-CT can detect gouty tophi and bone marrow edema, further supporting accurate diagnosis [27, 28]. CT has also been shown to have a greater capacity at identifying differential diagnoses than MRI in the context of peripheral arthritis [29].

Previous studies have highlighted the utility of iodine maps in detecting soft tissue inflammation in peripheral arthritis. However, they have not established a recommended post-contrast scan timing. Ulas et al. described that contrast-enhanced DE-CT techniques and MRI perform equally well in arthritis imaging; they utilized a 180-s scan after contrast administration [26]. Diekhoff et al. also applied a 180-s model and concluded that ultra-low-dose CT imaging allows for the detection of soft tissue inflammation [29]. Fukuda et al. used a shorter 120-s protocol to prove that DE-CT is suitable to detect inflammatory activity in PsA patients [7]. Kayama et al., who demonstrated that the DE-CT iodine map is a valuable tool for quantitative assessment of therapeutic response in PsA, also utilized 120-s post-contrast measurements [30].

**Fig. 5:**
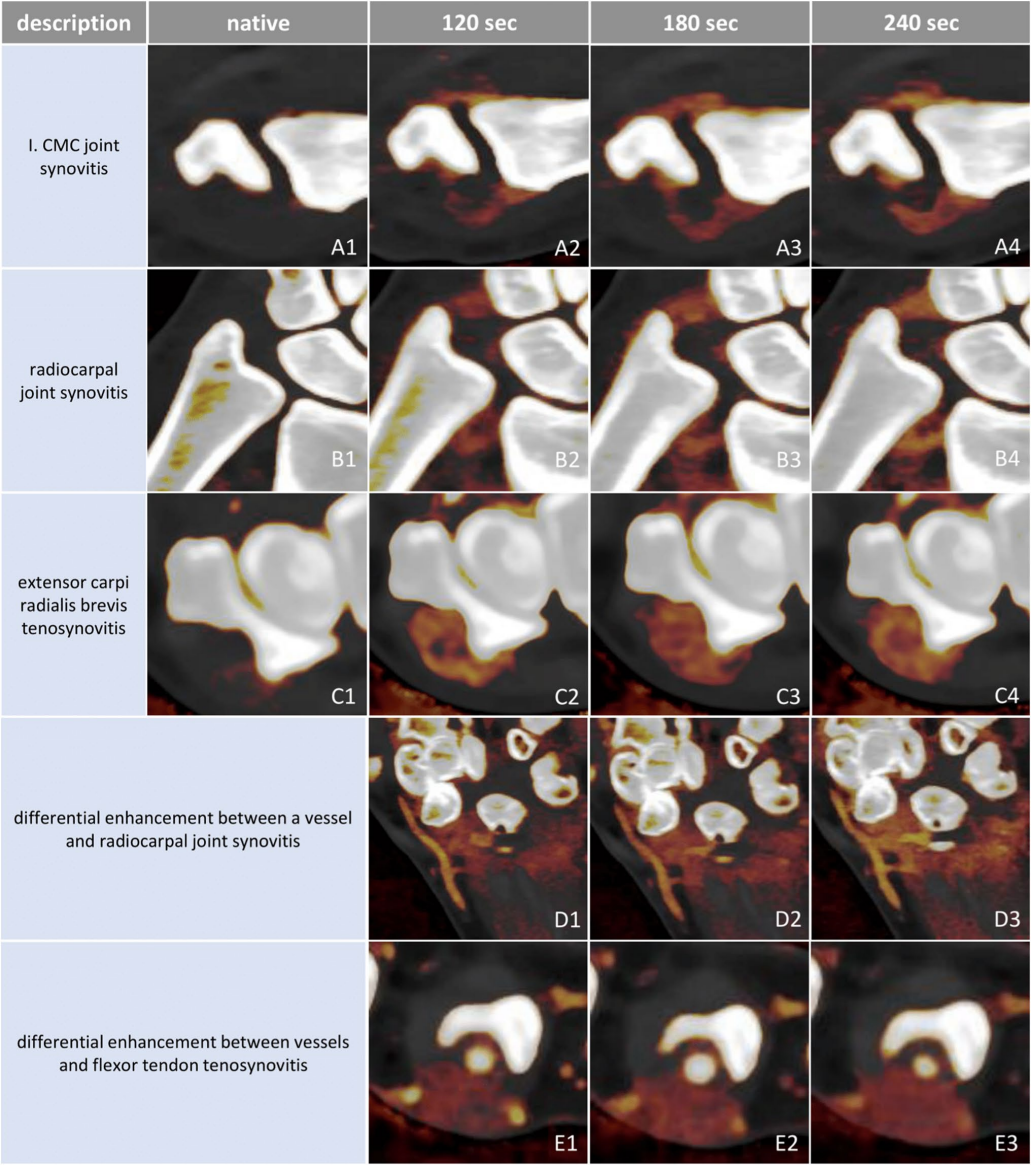
Appearances of enhancing inflamed synovial and tenosynovial structures, as well as contrast in enhancement between vessels and inflamed tissues across native and investigated post-contrast phases seen on PCD-CT iodine maps. In the native phase, inflamed synovial and tenosynovial structures show no enhancement (**A1**,** B1**, **C1**). As for the post-contrast phases, they display very similar enhancement intensities and spatial distributions of inflammation across each phase. The apparent differences in enhancement observed between the 240-s phases and the earlier ones are due to the normalization of the iodine map’s color coding to an enhancing vessel, which gradually washes out. This effect can be corrected by manually adjusting the windowing, enabling more or less color to be attributed to the same iodine concentration values. The greatest contrast between enhancing vessels and inflamed tissues was seen in the 120-s phase, as demonstrated by rows **D** and **E**. CMC, carpometacarpal; PCD-CT, photon-counting detector CT; sec, second

Here, we analyzed for the first time the optimal time point for post-contrast spectral PCD-CT scanning to visualize inflammatory activity in arthritis patients. We found no significant difference (*p* > 0.05) in the enhancement values of inflamed synovial, tenosynovial, and periungual soft tissues across the investigated (120, 180, 240 s) phases after intravenous iodine contrast administration (Fig. 3A, Supplementary Table 1). Inflamed tissues appeared equally enhancing on all post-contrast phases (Fig. 5A–C rows). The apparent differences in enhancement noticeable in Fig. 5 were due to the normalization of iodine maps to blood vessels, which showed a gradual decrease in contrast concentration with each subsequent phase. This effect could be eliminated by adjusting the windowing of the color map.

Synovial neoangiogenesis can be observed in immune-mediated arthritis. Because of the enhanced, abnormal type of vascularization, vasopermeability increases [31, 32]. Potentially, this phenomenon contributes to the increased diffusion and slow washout of contrast agent into the inflamed synovial tissues. No specific studies were published about this phenomenon before. Based on CE MRI measurements, synovial enhancement reached its maximum 12 min after intravenous contrast administration [33].

When comparing the enhancement of interest structures to that of control structures, the 120-s post-contrast phase provided the greatest contrast between enhancing blood vessels and inflamed structures (Fig. 5D and E rows). This can be explained by the observation that while enhancement values in inflamed structures remained stable, vascular enhancement gradually decreased, approaching that of inflamed tissues. In our experience, this phenomenon may aid the evaluation of subtle inflammation, where distinguishing inflamed synovium from adjacent small arteries can be challenging. It could enhance diagnostic confidence in early disease and support more objective assessment of treatment response, particularly in small distal joints. Muscle tissue and fat exhibited the same differentiation from interest structures across all post-contrast phases.

All interest and control structures maintained the same mean density across the native and the three post-contrast phases. This suggests that our measurement points were consistently positioned in the same locations across all phases (Fig. 3B).

The anatomical distribution of inflamed structures in our patients aligned with their diagnoses. Our work affirms previous studies that iodine mapping is a useful tool in detecting inflammatory activity in arthritis patients [6, 7, 29, 30].

The most important concern when using CT is radiation exposure. With low-dose CT protocols, the effective dose is often comparable to conventional radiography. PCD-CT allows for the simultaneous acquisition of both regular and spectral CT data, enabling detailed assessment of osseous structures and iodine mapping. This technique offers reduced effective dose values compared to conventional energy-integrating detector (EID) CT. An added value of the investigated PCD-CT technique is that it significantly reduces the impact of beam hardening artifacts compared to conventional CT, and high- or even ultra-high-resolution imaging is also available. In addition, low-energy photons contribute more to the image contrast in PCD-CT, upgrading the image contrast-to-noise ratio of iodine-based contrast materials. The effective dose value in our study was 0.028 mSv for each post-contrast phase. If just one post-contrast phase is utilized, like we concluded, further dose reduction could be achieved.

**Fig. 6:**
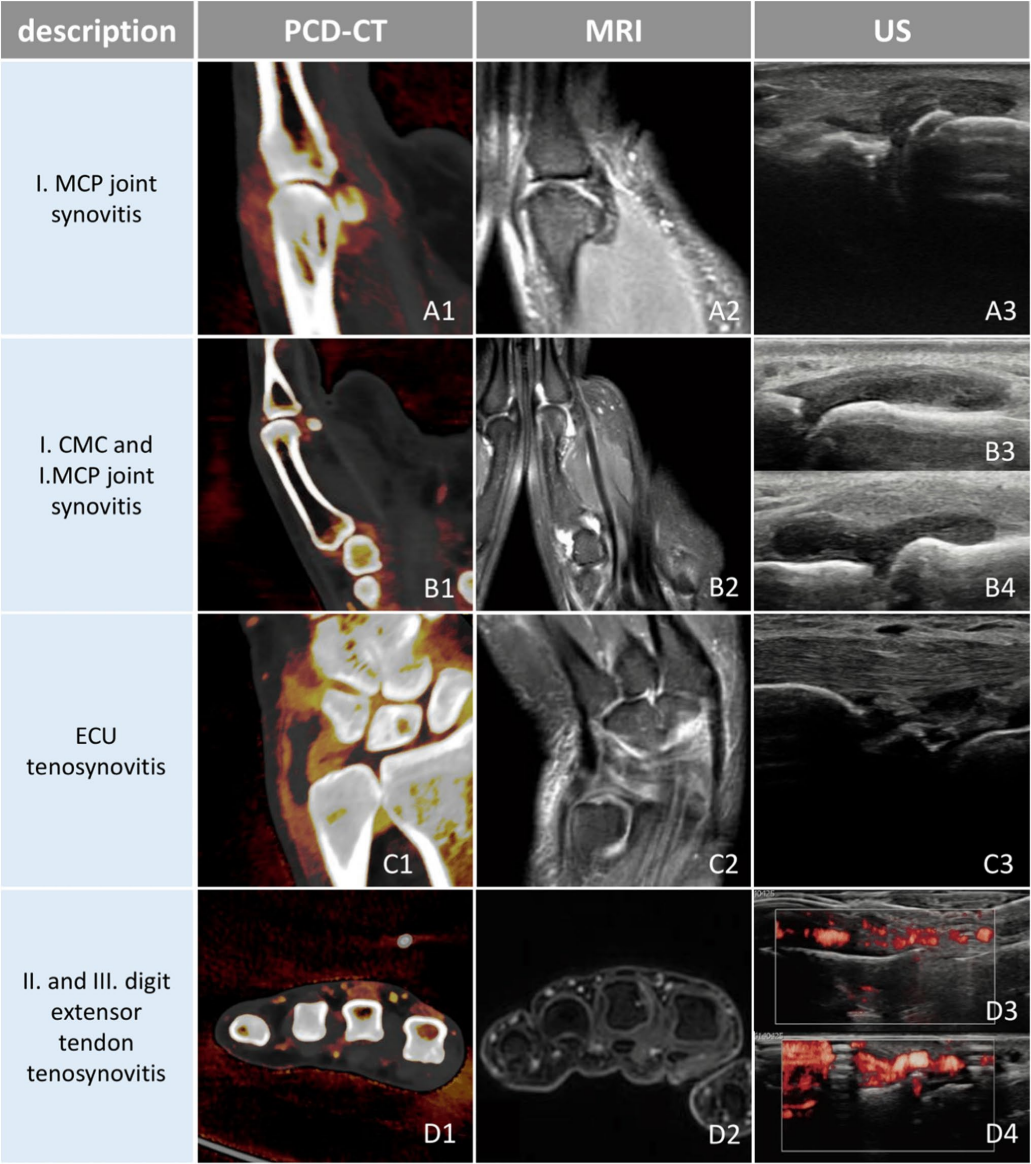
Comparisons of common pathologies in the same patients across different modalities used in the investigation of peripheral arthritis. On PCD-CT iodine maps, synovitis and tenosynovitis appear as areas of thickened synovium and tenosynovium with high iodine concentration, represented in red on the color map. On PD MRI sequences (**A2**,** B2**, **C2**), these pathologies present as high-signal fluid within the joint capsule or around the tendon, thickened synovium with intermediate signal intensity, and enhanced contrast uptake on post-contrast sequences (**D2**). In some cases, inflammation is more conspicuous on iodine maps than on contrast-enhanced MRI, particularly in distal digital structures, due to higher spatial resolution, improved contrast, and reduced motion artifacts (**D1** and **D2**). Ultrasound reveals intraarticular and peritendineal fluid accumulation, thickened synovium, and hypervascularity, which is highlighted on Doppler imaging. PCD-CT iodine maps provide a comprehensive and standardized overview of the affected hand while delineating inflammation with great contrast and high resolution, minimizing study duration and artifacts and allowing for the sensitive detection of bony changes at the same time. CMC, carpometacarpal; ECU, extensor carpi ulnaris; IP, interphalangeal; MCP, metacarpophalangeal; MRI, magnetic resonance imaging; PCD-CT, photon-counting detector CT; PD, proton density; US, ultrasound

All in all, CT is a quick, standardized procedure that enables high-resolution 3D imaging. It could serve as a good alternative for those who cannot bear the lengthy examination times or who are contraindicated for MRI and is generally the preferred modality by patients [34]. Figure 6 compares the appearances of common pathologies in peripheral arthritis across PCD-CT iodine maps, MRI, and ultrasound, as observed in our patients.

The limitations of our study were the size of the cohort, the single-center study design, and the lack of a histopathologic reference standard, as well as a healthy control group. Also, no contrast-enhanced MRI and CT comparisons were made. To extend our observations, the comparison of different DE-CT and PCD-CT protocols could serve valuable information. Also, the comparison of asymptomatic volunteers to symptomatic rheumatological patients could provide substantial data but raise ethical questions. A follow-up study would also yield valuable results.

To conclude, spectral PCD-CT imaging is a fast and comprehensive imaging method to analyze peripheral inflammatory activity in immune-mediated arthritis, while radiation exposure remains low when peripheral joints are imaged. CE PCD-CT measurements could help clinical decision-making—such as improving early diagnosis, guiding treatment selection, or reducing unnecessary follow-up imaging.

Our study found that the enhancement of inflamed joints, tendons, and periungual soft tissues in peripheral arthritis remains stable between the 120- and 240-s delay phases, indicating the need for broader time points to further characterize the dynamics of inflammatory enhancement. However, the 120-s delay phase provided the greatest contrast between inflammation and surrounding vascular structures, which may enhance diagnostic confidence in more subtle cases. Evaluating later phases may reveal greater contrast between inflamed tissues and muscle, potentially further improving diagnostic certainty.

## Supplementary Information

Below is the link to the electronic supplementary material.

ESM 1 (DOCX 21.1 KB)

## Data Availability

If needed, raw data, datasheets, and statistical analysis of the abovementioned work could be provided.

## Abbreviations

ACR: American College of Rheumatology
cc: Concentration
CMC: Carpometacarpal
CRP: C-Reactive protein
CT: Computed tomography
DAS28: Disease activity score
DE-CT: Dual-energy CT
DIP: Distal interphalangeal
DRU: Distal radioulnar
ECRL: Extensor carpi radialis longus
ECRB: Extensor carpi radialis brevis
ECU: Extensor carpi ulnaris
ESR: Erythrocyte sedimentation rate
EULAR: European Alliance of Associations for Rheumatology
FCU: Flexor carpi ulnaris
IP: Interphalangeal
MCP: Metacarpophalangeal
MRI: Magnetic resonance imaging
PCD-CT: Photon-counting detector CT
PD: Proton density
PIP: Proximal interphalangeal
PST: Periungual soft tissue
ROI: Region of interest
sec: Second
STT: Scaphotrapeziotrapezoidal
tDLP: Total dose length product
US: Ultrasound
