# Supplementary material for: Optimizing contrast timing in photon-counting detector CT for the assessment of peripheral arthritis
Source: Skeletal Radiol. 2025 Aug 8;54(11):2565–78. doi: 10.1007/s00256-025-04993-4 (PMC12460460; doi:10.1007/s00256-025-04993-4)
Supplement: Supplementary file 1 — (DOCX 21.1 KB) [file 256_2025_4993_MOESM1_ESM.docx]

**Supplementary Tables**

**Supplementary Table 1.**

|  | p values - density | p values -concentration |
| --- | --- | --- |
| 120 sec vs 180 sec | 0.899 | 0.593 |
| 120 sec vs 240 sec | 0.899 | 0.899 |
| 180 sec vs 240 sec | 0.899 | 0.653 |

**Supplementary Table 1.** P values from Tukey post hoc pairwise comparisons of contrast-enhanced phases (120, 180, 240 sec) for density and iodine concentration values. No statistically significant differences were observed between the phases (significance threshold: p < 0.05).

**Supplementary Table 2.**

| **contrast** | **time** | **estimate** | **lower.CL** | **upper.CL** | **p.value** |
| --- | --- | --- | --- | --- | --- |
| vessel - inflammation | 0 | -0.20263 | -0.30779 | -0.09748 | < 0.0001 |
| muscle - inflammation | 0 | 0.19577 | 0.09062 | 0.30092 | < 0.0001 |
| fat - inflammation | 0 | -1.40902 | -1.51417 | -1.30387 | < 0.0001 |
| vessel - inflammation | 120 | 1.05460 | 0.94945 | 1.15975 | < 0.0001 |
| muscle - inflammation | 120 | -1.09434 | -1.19949 | -0.98918 | < 0.0001 |
| fat - inflammation | 120 | -2.94487 | -3.05002 | -2.83972 | < 0.0001 |
| vessel - inflammation | 180 | 0.64851 | 0.54336 | 0.75366 | < 0.0001 |
| muscle - inflammation | 180 | -1.08606 | -1.19121 | -0.98091 | < 0.0001 |
| fat - inflammation | 180 | -2.98819 | -3.09334 | -2.88304 | < 0.0001 |
| vessel - inflammation | 240 | 0.36416 | 0.25901 | 0.46931 | < 0.0001 |
| muscle - inflammation | 240 | -1.03531 | -1.14046 | -0.93016 | < 0.0001 |
| fat - inflammation | 240 | -2.98743 | -3.09258 | -2.88228 | < 0.0001 |

**Supplementary Table 2.** Estimated differences in iodine concentration and corresponding 95% confidence intervals between inflamed and control structures (vessel, muscle, fat) across different phases (native, 120, 180, 240 sec), based on a linear mixed-effects model. Statistically significant differences were observed between inflamed and control tissues across all phases (p < 0.0001).

**Supplementary Table 3.**

**Supplementary Table 3.** Estimated differences in density and corresponding 95% confidence intervals between inflamed and control structures (vessel, muscle, fat) across different phases (native, 120, 180, 240 sec), based on a linear mixed-effects model. Statistically significant differences were observed between inflamed and control tissues across all post-contrast phases (p < 0.0001).

| **contrast** | **time** | **estimate** | **lower.CL** | **upper.CL** | **p.value** |
| --- | --- | --- | --- | --- | --- |
| vessel- joint | 0 | -8.9460 | -11.38106 | -6.5110 | 0.00000 |
| muscle - joint | 0 | 1.7918 | -0.64329 | 4.2268 | 0.21846 |
| fat - joint | 0 | -128.6491 | -131.08419 | -126.2141 | 0.00000 |
| Vessel - joint | 120 | -8.6866 | -11.12167 | -6.2516 | 0.00000 |
| muscle - joint | 120 | 1.3671 | -1.06794 | 3.8022 | 0.44856 |
| fat - joint | 120 | -126.7042 | -129.13922 | -124.2691 | 0.00000 |
| vessel - joint | 180 | -9.5373 | -11.97237 | -7.1023 | 0.00000 |
| muscle - joint | 180 | 2.1462 | -0.28886 | 4.5812 | 0.10227 |
| fat - joint | 180 | -124.4788 | -126.91386 | -122.0438 | 0.00000 |
| vessel - joint | 240 | -9.7155 | -12.15055 | -7.2804 | 0.00000 |
| muscle - joint | 240 | 1.8233 | -0.61172 | 4.2584 | 0.20533 |
| fat - joint | 240 | -124.0118 | -126.44683 | -121.5767 | 0.00000 |

**Supplementary Table 4.**

| **Concentration** |  | **Confidence interval** | **P value** |
| --- | --- | --- | --- |
|  | RA | -0.25 – 0.12 | 0.485 |
|  | PsA | -0.20 – 0.21 | 0.957 |
|  | SpA | -0.32 – 0.26 | 0.810 |
| **Density** |  |  |  |
|  | RA | -5.86 – 9.26 | 0.646 |
|  | PsA | -5.71 – 11.21 | 0.508 |
|  | SpA | -11.38 – 10.75 | 0.953 |

**Supplementary Table 4.** Estimates and 95% confidence intervals from a linear mixed-effects model assessing iodine concentration and density in relation to diagnosis (RA, PsA, SpA). No significant differences in iodine concentration or density were observed between diagnostic groups.

**Supplementary Table 5.**

|  | **density** | **density** | **concentration** | **concentration** |
| --- | --- | --- | --- | --- |
|  | **R value** | **p value** | **R value** | **p value** |
| **age** | 0.12 | 0.56 | 0.13 | 0.61 |
| **CRP** | -0.1 | 0.96 | -0.2 | 0.94 |
| **ESR** | -0.05 | 0.98 | -0.03 | 0.89 |
| **Severity index (DAS28)** | -0.01 | 0.95 | -0.01 | 0.96 |
| **Disease onset** | -0.05 | 0.80 | -0.07 | 0.86 |
